# Covid-19 Response From Global Makers: The Careables Cases of Global Design and Local Production

**DOI:** 10.3389/fsoc.2021.629587

**Published:** 2021-03-18

**Authors:** Barbara Kieslinger, Teresa Schaefer, Claudia Magdalena Fabian, Elisabetta Biasin, Enrico Bassi, Ricardo Ruiz Freire, Nadine Mowoh, Nawres Arif, Paulien Melis

**Affiliations:** ^1^ Centre for Social Innovation, Technology and Knowledge, Vienna, Austria; ^2^ Katholic University Leuven, Centre for IT & IP Law–imec, Leuven, Belgium; ^3^ OpenDot, Milan, Italy; ^4^ Faculty of Administration Sciences, University of Pernambuco, Brazil; ^5^ Mboalab Biotech, Yaounde, Cameroon; ^6^ Science Camp, Basrah, Iraq; ^7^ Waag Society, Amsterdam, Netherlands

**Keywords:** COVID-19, makerspace, social innovation, open source hardware, DIY healthcare

## Abstract

Makerspaces—informal shared spaces that offer access to technologies, resources and a community of peer learners for making—across the globe initiated a rapid response to the lack of medical hardware supplies during the global pandemic outbreak in early 2020 caused by the Corona virus (COVID-19). As our health systems faced unexperienced pressure, being close to collapsing in some countries, and global supply chains failing to react immediately, makers started to prototype, locally produce and globally share designs of Open Source healthcare products, such as face shields and other medical supplies. Local collaboration with hospitals and healthcare professionals were established. These bottom-up initiatives from maker networks across the globe are showing us how responsible innovation is happening outside the constraints of profit-driven large industries. In this qualitative study we present five cases from a global network of makers that contributed to the production of personal protective equipment (PPE) and healthcare-related products. We draw our cases from the experiences made in *Careables*, a mixed community of people and organizations committed to the co-design and making of open, personalized healthcare for everyone. With the presented cases we reflect on the potential implications for post-pandemic local production of healthcare products and analyze them from a social innovation perspective. These global experiences are valuable indications of transformative innovations that can reduce dependencies from international supply chains and mainstream mass production.

## Introduction

“Makerspaces are informal shared spaces located in communal, educational and increasingly also commercial settings, which provide their members with access to technologies, resources and most importantly a community of peer learners for making” (Ahmadi et al., 2019).

During the rapid spread of the novel Coronavirus (COVID-19) worldwide, which puts our health systems under unexperienced pressure and brings them close to collapsing in some countries, we are all witnesses to the importance of the maker community for a rapid response to the lack of medical hardware supplies (Ranney et al., 2020). Across the world we see initiatives popping up where makerspaces are called to use their digital fabrication tools to, e.g., 3D print valves for life-saving Coronavirus treatments or face shields to offer some protective gear for doctors (Diez and Baeck, 2020). But not only does the maker community contribute to the rapid production of needed pieces, it also shows its responsible innovation capacities by rapidly prototyping, testing, documenting, and reproducing new products that are needed in times of this pandemic, such as hands-free 3D-printed door openers to help against the spread of Coronavirus. Medical Hackathons are organized around the globe to design and deploy Open Source Hardware (OSH) medical products.

However, this first aid response of the maker community does not go without friction, especially when dealing with critical medical equipment that needs to adhere to strict quality control and standards and represents a large business field for companies specialized in this area. One of the first instances of such a conflict appearing in international media was the case of a volunteer maker in Italy, who produced 3D-printed valves for life-saving Coronavirus treatments. The original manufacturing company refused to release the design files for the valves, forcing the volunteer maker to reverse-engineer the valve (Peters, 2020). The ethical question that remains to be answered in this case is whether the original manufacturer did not release the original files due to a concern of quality or due to a business-driven motivation. The great concern for quality standards is shared across the maker community and the rapidly established working groups and testing spaces with doctors. Makers are working with medical review teams to validate the utility and safety of new solutions quickly, before entering them into Open Source Hardware collaboration and hosting platforms (Brown, 2020).

The bottom-up initiatives from maker networks across the globe are currently showing us how responsible innovation is happening outside the constraints of profit-driven large industries. We are witnessing critical, socially responsible making these days and a professionalization of the maker-driven open hardware movement that is comparable to Open Source Software which is running the world nowadays. But is the maker community putting social interests before business interests? What effects will the Open Source hardware designs, that are currently being created and shared, have on the future of manufacturing? Will we see new collaborations across established industries and makers emerging? How will this affect society and especially the younger generation? These are just some of the emerging questions that science and technology studies in a joint effort of different disciplines still have to address.

In this paper, we build on the experiences made during the COVID-19 pandemic by a small number of globally distributed makerspaces and fablabs. We aim to provide rich descriptions of the makers’ COVID-19 response and reflect on their potential wider societal implications in the future. The main objective of this study is to critically reflect from within the maker community on the crisis response actions taken, showing current challenges and limitations as well as offering a stimulus for further analysis of the transformative character of makerspaces. We have chosen a case study approach as in qualitative research the complexity of each case provides us with an important context for understanding the issue we are studying (Flick, 2017).

The five selected cases have previously been active in open healthcare practices and have been loosely connected via *Careables*, a project dedicated to personalized open healthcare (www.careables.org). While these maker communities all vary in their COVID-19 response approaches, which we discuss in detailed case description, a focus group discussion revealed a series of commonalities amongst makers when it comes to scaling their activities, which we then related to the theories of social and transformative innovation theories.

## Do-It-Yourself (DIY) Healthcare, Makers and Health and Care Products

### Overview

Bottom-up digital social innovations are on the rise, including in healthcare. Over recent years we have witnessed a growing number of grassroots solutions in do-it-yourself (DIY) healthcare, including the development of Open Source hardware and DIY practices which may counteract current healthcare supply shortages. Via Open Source approaches communities can collaboratively improve and co-produce new solutions, in consultation with public health authorities (Richterich, 2020). Innovators, users of healthcare products, and communities in healthcare are starting to collaborate by using digital technologies to co-create knowledge and solutions for a wide range of needs. These solutions range from Open Source hand prosthetics, 3D printed writing tools to support kids with physical limitations, to add-ons for wheelchairs, and everything in between. If we look into the medical field, we see similar tendencies towards experimentation and creation of alternative solutions beyond the standardized practices, e.g., in the fields of biohacking, patient experimentation, and Open Source hardware for medical devices.

These community-led or civic innovations are responses to societal issues that cannot be met by our healthcare systems nor by industry. Criado, Rodriguez-Giralt and Mencaroni (2016) even position open design and participatory prototyping strategies in a more political context and stress the activist character when applied by the independent living movement in Spain. They relate the experiences of open prototyping with and by disabled people to the critical making notion defined by Ratto (2011), which stressed the learning aspects and the societal relevance of DIY activities in maker communities.

Closely related to these critical making properties is *Careables*, an initiative that is rooted in the context of personalized open healthcare development. It is a mixed community of people and organizations committed to the co-design and making of open, personalized healthcare for everyone driven by a set of underlying principles for responsible making. It started 2018 as a European funded innovation action under the Horizon 2020 program and has since grown to a worldwide community, mostly via a global network of social and technological innovators called Global Innovation Gathering (GIG). The *Careables* platform^1^ and its documentation repository on Welder-app^2^ currently registers over 180 open designs for open healthcare solutions, next to other resources, such as legal and ethical guidelines or training resources. *Careables* encourages care receivers, healthcare professionals, and makers to join forces and to co-create tailor-made solutions designed for supporting and better suiting the care receivers’ needs.

Further, the global network of fablabs recently launched Fab Care as a global initiative to support fablabs, makerspaces and hackerspaces which are working in assistive technologies, in creating personalized solutions for people with physical challenges to improve their quality of life.

Beyond the civic-innovation character, we also see more and more established healthcare institutions, such as hospitals, therapeutic and care centers, starting to work with digital fabrication tools. In some hospitals, makerspaces are already part of their infrastructure (Marshall and McGrew, 2017). While these initiatives are less driven by activism or socially driven innovation needs, they equally recognize the values of local on-demand production of spare parts in healthcare equipment, therapeutic devices, creativity, and innovative prototyping. In these health makerspaces medical staff find access to tools, materials, and the required knowledge to test new ideas and build prototypes. With the experiences of the momentary personal protective equipment (PPE) and other medical device shortage during the COVID-19 crisis on the one hand and digital fabrication tools and skills on the rise on the other, we may experience a growing penetration of a demand for local production in healthcare.

These developments obviously bring legal and ethical issues to the table such as do-it-yourself solutions that may not always comply with medical standards and regulations. Problems may range, for example, from intellectual property law (e.g., see the above-mentioned case of the 3-D printed valve) to safety and specific laws for medical devices and PPE. Part of these problems arises, as in most cases product laws are aimed at large organizations rather than small entities. Makerspaces and these new forms of collaboration blur the classic hierarchical dichotomy between producers and consumers (Daly, 2016; Kamenjasevic and Biasin, 2018) and result in greater problems in ensuring the legal compliance of the co-designed and co-created products.

### Maker’s COVID-19 Response Initiatives

In early 2020, when the COVID-19 pandemic had completely turned into the globally dominating health concern, bringing the health systems in many countries to their absolute limits, the reaction of the maker movement was instantaneous. Maker communities around the globe have been very active during the first wave of the COVID-19 crisis by responding to the shortage of PPE and other medical and healthcare-related products. One of the larger civic response communities is the Open Source Medical Supplies (OSMS)^3^. Initiated by Gui Cavalcanti, the founder and CEO of a robotics company, OSMS launched in March 2020 as a Facebook group, and rapidly brought together a global network of over 70,000 makers, fabricators, community organizers, and medical professionals in 55 countries collaborating on the unprecedented medical supply challenges caused by the COVID-19 pandemic. In their global impact dashboard, the network currently indicates that over 16 Million supplies have been delivered by the global community, with face shields being by far the most frequently produced device.

The variety of PPE and medical supplies that have been produced in these collective networks are said to include around 50 different products, ranging from door openers, and ear savers to intubation boxes. These PPE serve as a means to reduce the spread of the virus following the available evidence that the virus is transmitted via air droplets when in close contact with infected persons and not air-borne. Therefore by providing equipment that supports frequent and effective handwashing or acts as disinfectants, helps preventing contact with droplets or helps avoiding contact with contaminated surfaces like door handles, an effective preventive measure is being taken especially in healthcare and community settings. The knowledge and research done by the OSMS global network have been documented and shared in case studies, community stories, a project library that gives access to many open designs, a map to find local response groups, and the Open Source Medical Supply Guide (Open Source Medical Supplies, 2020). Also, the *Careables* community shifted its focus of activities from supporting DIY healthcare for people with disabilities to collecting, documenting and sharing information and Open Source solutions to fight COVID-19. The *Careables* COVID-19 collection currently includes around 50 Open Source hardware projects, ranging from different versions of face masks and shields to intubation boxes and door openers. In addition, background information and legal guidance on the responsible production and use of DIY products are shared with the maker community worldwide.

According to a survey done by the Fabfoundation (Fabfoundation, 2020), which was answered by 42 fablabs around the world, more than half of the 43 products made by the responding fablabs as a reaction to the COVID-19 crisis were locally approved or medically reviewed by an agency or organization. These include hospitals (for 22 products), healthcare professionals (for 15 products) or national (for 6 products) and local (for 2 products) healthcare institutions. The same report stresses the local context where most of the work happened to serve small local organizations in need. The authors of the study conclude that “A locally sourced, globally distributed manufacturing process could continue to fill an immensely important role in the months (and years) to come” (Fabfoundation, 2020, p.13).

Not only has the failure of global manufacturing supply chains accelerated the makers’ response; another important factor triggering the community action has been the financially underserved and fragile healthcare systems in many countries. In the United Kingdom an analysis of the maker response to COVID-19 pandemic by Richterich (2020) clearly establishes a link between the national austerity politics and the strained healthcare system. When relating the makers’ DIY production of healthcare equipment to the critical making theory of Ratto (2011) there is also a political dimension coming into play, as volunteers in makerspaces reacted to a governmental failure in healthcare supplies (Richterich, 2020).

Overall, the maker COVID-19 response initiatives strongly relied on the sharing of open designs and a self-organized production and dispatchment of the DIY equipment, via online and social media (Corsini et al., 2020; Zastrow, 2020). When a certain material was not locally available alternatives were explored, either by modifying the design, adapting material that was already available or hacking different parts of the product design (Fabfoundation, 2020). Richterich (2020) stresses this synergy of Open Source product design, it’s re-use, it’s adjustment, and it’s local production as an open hardware product as a core characteristic of the maker communities’ COVID-19 response.

## Social and Transformative Innovation Theory and Societal Implications of DIY Open Healthcare

The volunteer-driven, self-organized activities of the maker community have significantly contributed to the response phase of the COVID-19 pandemic and also drew attention to the latent innovation potential of the general public (Corsini et al., 2020). Since the rapid emerging of local makerspaces, hackerspaces, fablabs and the calling out of a global maker movement (Dougherty, 2012) experts have assigned this new culture of local manufacturing certain social transformation power (Diez, 2012; Smith, 2017; Millard et al., 2018; Bosse et al., 2019; Unterfrauner et al., 2020). The technological innovations advancing the manufacturing capacities of digital fabrication tools have offered a wide range of possibilities for social and community action. As Ruiz Freire et al. (2019) exemplify with the three-dimensional additive printers, that are nowadays accessible on the home market, technological innovations can lead to strong social, environmental, economic, and political implications. Based on a bibliographic analysis of innovation processes, the authors argue to regard social and technological innovations not as separate phenomena, but rather to consider innovations in their social, technical, economic, educational, and political realm (Ruiz Freire et al., 2019). The examples of DIY open healthcare production that we have discussed above need to be analyzed in a multilayered perspective as well.

Smith (2017), who studies makerspaces as sites for democratizing innovation activity, assigns them social innovation potential. He describes them as *socially transformative, educationally useful and entrepreneurially promising.* They offer capabilities for participation, deliberation and community development, which constitute their transformational and democratic potential. At the same time makerspaces also reproduce dominant values of society and the global economy, e.g., when they follow an open innovation agenda that tries to leverage the makers’ creativity for global manufacturing and following prevailing economic growth business models. Thus, we are witnessing contradictory developments of makerspaces, where we have open spaces aiming for democratic transformations next to spaces that adhere to traditional market-driven models (Unterfrauner et al., 2020).

From a critical making perspective, this path of entering the business-as-usual chain is not the one to follow, and many makers striking a social transformative or democratic path have started a search for more participatory models of production generally, and for the healthcare sector specifically. DIY manufacturing processes based on Open Source design may be social innovations that respond to certain social needs, but they also require new collaborative and financial models. Ratto (2011) critical making values, such as the societal relevance of making and the potential for learning and gaining critical knowledge during this process of material work, indicate the social and transformative innovation potential of DIY open healthcare production in maker communities.

**FIGURE 1:**
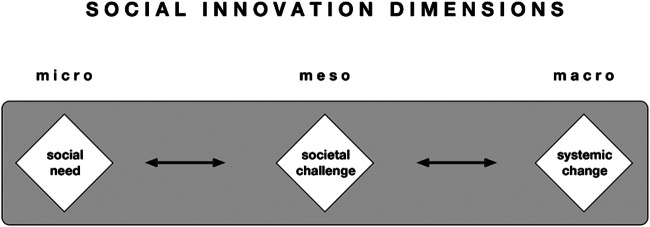
Adaption of the three Bureau of European Policy Advisers (BEPA) (2010) levels of social innovation.

As explained by social innovation theory, social innovations tackle social needs and respond to societal challenges (Holtgrewe and Millard, 2018). According to Bureau of European Policy Advisers (BEPA) (2010) societal levels model of social innovation, there are three interconnected levels, namely the social needs (micro level), the societal challenges (meso level), and the systemic change (macro level) (see Figure 1). At the micro level, social innovations are responding to local social demands, tackling specific problems on the ground that are not met by the market or public institutions. They respond in a bottom-up approach to the needs of particular groups, often including the beneficiaries themselves, such as vulnerable people. At the meso level, we see social innovations that are tackling societal challenges at large social scale or across whole sectors by combining social, economic, environmental, and cultural factors. It usually requires new forms of relations between actors, including adequate organizations, networks, and modes of collaboration for producing real and desired outcomes. At the macro level, social innovations generate system change. This can only happen when fundamental transformations in society are taking place, including a reform of underlying structures, changes in the relationships and powers in society. It often goes along with organizational and institution change, reforms of public policies, new governance arrangements and a changing mindset and cultures, allowing for more participation and empowerment. While this distinction of social innovations at the three levels is helpful for analysis it is also simplistic in a way, implying a somewhat linear view of society and possibly ignoring complex and unintended consequences (Holtgrewe and Millard, 2018). For the purpose of this work, it proves to be a useful instrument for discussing results of the makers’ experiences.

The DIY open healthcare activities of projects such as *Careables* are located at the social demand level, tackling specific problems of people, often from vulnerable groups, that are not addressed appropriately by the market or institutions. The COVID-19 PPE production started at a micro level, but with the enormous impact that the epidemic has on our social, political and economic systems, it also gets attention as a more societal challenge on a meso level. The third level, the systemic change or transformation level, requires fundamental changes in institutions, governance and policies. While in this analysis we will mostly stay at the micro level with the described cases, we want to explore how the actions and networks around the COVID-19 response of the maker movement may influence at meso and macro level, contributing to transformations in healthcare in the future.

## Methodology

As the overall methodology, a qualitative case study approach was chosen since it represents a versatile form of qualitative inquiry that is suitable for a comprehensive and in-depth investigation of complex issues and unclear boundaries (Harrison et al., 2017). Representatives of makerspaces from the *Careables* project and the GIG (Global Innovation Gathering) network volunteered to participate in this case study. They are listed as co-authors and are referred to in the following text as “case representatives,” sharing their insights and experiences through an interactive dialogue, guided by a self-reflection exercise and an online focus group discussion.

### Involved Case Representatives and Researchers

For the purpose of this study five makerspaces that have been very active during the COVID-19 crisis in very different contexts were invited to contribute to this research. Three of these five are members of the GIG network ^4^. It is a global network of social and technological innovators that aims to foster the sharing of knowledge and experience amongst members. Two makerspaces are partners of the *Careables* project consortium. In the following list we introduce the representatives of the makerspaces who contributed to this article.

- Brazil: Ricardo Ruiz Freire (Member of the GIG Supervisory Board)
- Cameroon: Nadine Mowoh (Member of the GIG network)
- Iraq: Nawres Arif (Member of the GIG Supervisory Board)
- Italy: Enrico Bassi (Director of the makerspace OpenDot^5^)
- The Netherlands: Paulien Melis (Programme Developer of the makerspace WAAG^6^)

In addition to the above-mentioned case representatives four researchers from the *Careables* research team participated in this research. Three of them are female academics at the Center for Social Innovation in Austria, bringing in interdisciplinary perspectives, with an academic background spanning the disciplines of psychology, sociology, pedagogy, and economy. One researcher is a female legal expert working for the KU Leuven Center for IT & IP Law in Belgium. This diversity in backgrounds was important to understand the complexity of the cases and to improve the integration of diverse perspectives through a series of discussions and reflections. The three researchers from the Center of Social Innovation were also the ones who designed this qualitative study and took the lead in analyzing the result.

In general, the overall culture of this research study was collaborative and cooperative, since no single researcher imposed their interpretation, and the results were additionally discussed with all contributors of the paper.

## Research Design

**FIGURE 2:**
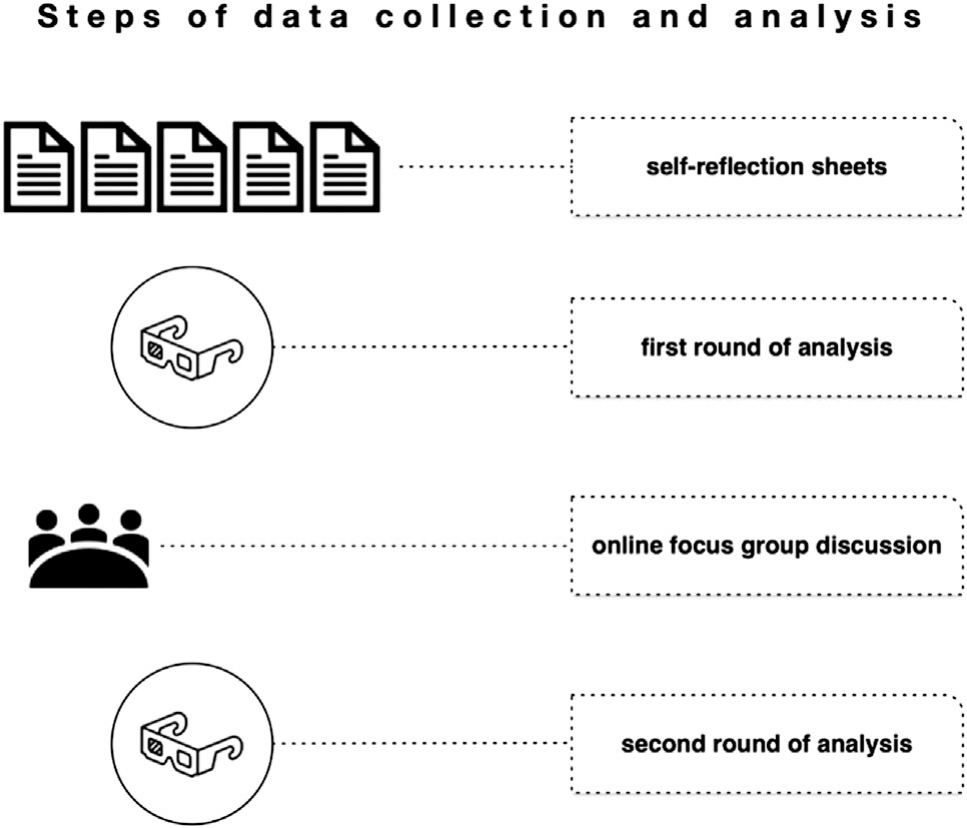
Steps of data collection and analysis.

Two data collection instruments were prepared to learn about and analyze the COVID-19 activities in the five cases: a self-reflection exercise and an online focus group discussion (Figure 2).

### Self-Reflection

For the collection of the data, the research team prepared a self-reflection exercise, which guided the case representatives in their self-reflection process. The self-reflection exercise was based on the following questions, which participants answered in relation to their the COVID-19 response activities of their makerspaces:

- *Case description:* Please describe what were/are your main activities of COVID-19 response. What motivated you to become active as COVID-19 responder? What partnerships/networks/collaborations have you established or are you making use of? How do/did you finance the production of PPE? Where do/did you get the designs from?
- *Perceived impact and achievements:* Please describe the perceived impact that you achieved so far with your activities and what has been the public/political perception of your activities. Has there been any public/political recognition of your contribution? You may also report on the impact achieved by other makers in your community.
- *Barriers and challenges:* Please reflect on the barriers and challenges that you encounter during your COVID-19 activities.
- *Future implications:* Please reflect on the future potential implications that you see from your experiences with regards to post-pandemic local production of healthcare products.

The self-reflection reports, which were filled in by the case representatives during a 3-weeks period in October 2020, were analyzed by the research team in a first round and provided the ground for the structure of the online focus group discussion.

### Online Focus Group

The online focus group was organized on November 3, 2020 via the videoconferencing platform ZOOM and lasted 75 min. Representatives from the cases in Brazil, Cameroon, and Italy, as well as the four researchers introduced above, took part and aimed at elaborating a deeper understanding of the described cases and future scaling options.

**FIGURE 3:**
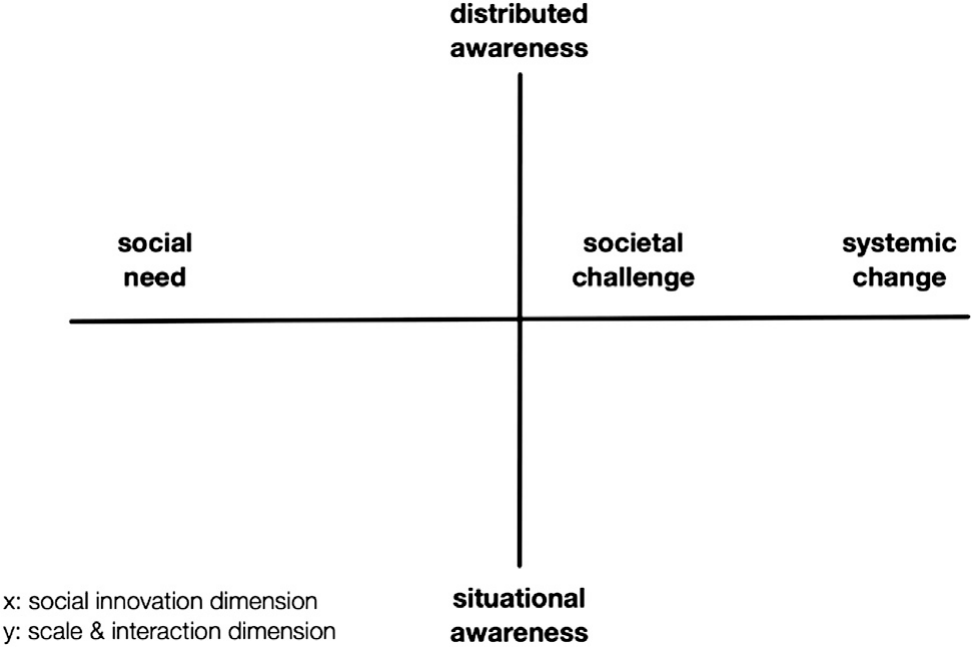
Scale and social innovation matrix (adapted from Unterfrauner et al., 2020).

The starting point of the focus group was a short presentation by the researchers of the three dimensions of social innovation also shared on a Google Jamboard-board and presented in Chapter 3 of this article. After this introduction, the case representatives were invited to place their COVID-19 response initiatives on the respective dimension of social innovation (micro—meso—macro) and explain their decision. In the next step, a second dimension was introduced, the scale and interaction dimension, as an indicator for connectedness (Figure 3). This dimension on the vertical axis refers on the one end to a situational awareness, where single actors tend to work relatively isolated, unconnected, and focus on the local area working on very local issues. On the other end of the axis, we speak about distributed awareness, referring to very strongly networked, interconnected makes, who work collaboratively over large areas, or even globally. This matrix of scale and social innovation dimension has been applied in previous studies and has provided valuable insights into the characteristics of maker initiatives (e.g., Unterfrauner et al., 2020).

The case representatives placed again their COVID-19 response initiatives on the respective x- and y-axes and discussed their positioning with the group. After this introduction the core discussion focused on two questions:

- Do case representatives wish that their COVID-19 activities in the different countries scale from micro, to meso, to macro level?
- If not, why not? If yes, under which conditions?

One of the leading researchers facilitated the discussion, while the second one summarized the main aspects of the discussion on the Jamboard to visualize the key points discussed. The third researcher took additional notes. The discussion was moderated to make participants reflect on their cases and to gain themselves new insights into their specific situation. In qualitative research the researcher is not necessarily the invisible neutral, but may also contribute to a moral discourse and spark transformative processes (Flick, 2018). The online focus group was also audio recorded based on the informed consent given by participants, as a basis for the later analysis.

### Analysis and Presentation of Data

The analysis of the case studies is based on the self-reflection reports of the case representatives and the summary of the online focus group discussion. Qualitative content analysis according to Mayring (2014) was selected as a suitable approach for this explorative study. It comprises a holistic and subjective procedure that is used to interpret and categorize qualitative data. This analytical process makes sense of the data, it describes and highlights important findings and allows to draw clear links between the research objectives and the summary findings. The conventional approach to content analysis (Hsieh and Shannon, 2005) was used here, where researchers avoid using pre-conceived categories, allowing the categories to emerge from the data.

The research material was analyzed through two iterative phases, from October 29, 2020 to November 11, 2020 by the three female researchers from the Center for Social Innovation, who also figure as the first authors of this manuscript. Data were analyzed both inductively and deductively in these two phases. Specifically, two forms of data analysis triangulation were carried out by the researchers to ensure a rigorous and robust approach (Leech and Onwuegbuzie, 2007). First, the self-reflection reports were coded individually by each researcher independently and provided insights about the research material that were shared and discussed. Preliminary codes were then agreed and grouped into sub-codes. The findings from this first coding experience also served as a basis for the focus group discussion, where they were critically reflected with the case representatives. In the second phase of the analysis the original codes were applied to the summary text of the focus groups, first individually by each researcher and then discussed jointly. At the end of this process the following codes, sub codes and a final grouping of the codes was agreed (Table 1).

**TABLE 1:** Overview of codes, sub codes, and their grouping

Original codes	Sub-codes	Grouped codes
Partnerships	Collaboration	Networks, partnerships, collaborations
Networks (local, regional, National, global)		
Collaboration with healthcare professionals		
Collaboration with educational sector		
Relationship with healthcare sector		
National and international collaboration		
Collaboration with companies and industry		
Collaboration With specific target groups, e.g. police, army		
Coordination of local groups	Coordination	
Coordination of national activities		
Decreasing visibility in large networks	Challenges in networks	
Increased complexity of structured networks		
Not losing contact to local communities		
Lacking Cooperation with government		
Local Context adaption	Local embedding	
Local aspects		
Local coordination groups		
Local needs		
Scaling	Scale	Value, scale, infrastructures
Upscaling		
Shared principles, values	Values	
Voluntary contributions, volunteers		
Political support		
Power of networks		
Fexibility		
Trust		
Sustainability	Sustainability	
Business models		
Funding		
Platforms	Infrastructures	
Local infrastructure		
Communication		
Logistics		
Local supply chains		
National coordination		
Structured Networks		
Overcoming barriers in the production (material, legal and ethical aspects)	Challenges of infrastructures	
Lack of resources, e.g. material		
Lack of funding		
Sensitization	Awareness	Education, training, skills, awareness
Local and international awareness		
Education	Education	
Skills, experiences		
Exchange of knowledge		
Tackeling misconceptions		
Guidance	Training	
Training		
Empowerment of citizens	Skills	
Pushing maker skills towards other domains, e.g. health-sector		
Critical thinking of negative impact		
Safety	Safety	Safety, quality, legal aspects
Quality, certification	Quality	
Prototyping and testing		
Legal aspects (National and International)	Legal aspects	

We are aware that this study is exploratory in nature and is giving a rich qualitative view on the reported cases, but is limited in its scope. This is the very nature of qualitative research. Engaging the case representatives in a reflective focus group discussion and being part of the authoring team might shed some doubt on the validity of this research. However, we believe that by doing so we have guaranteed the authenticity of the experiences and our findings are internally coherent. In particular the focus group discussion has helped to reflect on the constructs emerging from the first analytical phase and has allowed the three main researchers to capture data “from the inside” and gain a deeper understanding of the phenomena under discussion (Miles and Huberman, 1994). This transdisciplinary approach was important for a meaningful knowledge co-production as described by Thompson et al. (2017). The integrative and participatory processes during the self-reflection reporting and the focus group discussion opened the complex context of the maker movement for the main researchers. The collaboration of the actors involved in this study, who transcend disciplinary and academic boundaries, has been growing over the years and accumulates in this study. We belief that a certain level of mutual trust amongst all co-researchers is important for the transdisciplinary co-production (Thompson et al., 2017), leading to socially robust knowledge in the sense of Nowotny et al. (2001).

The following case descriptions represent summaries of the self-reflection reports while chapter 6 presents the results from the online focus group.

## Case Descriptions

The following five cases of maker responses to COVID-19 cover different perspectives and contexts. Some focus on their lab’s activities, some relate more on the national activities all together, and overall, they give a good representation of the diversity of actions encountered in makerspaces across the globe.

The Brazilian Case (LabCOCO, Casa Criatura, Coletivo 3D and LabProComum; Olinda)

In Brazil, before the outbreak of the pandemic, four maker-oriented organizations had established collaborations with the *Careables* project and started to function as local hubs which connect local communities of persons with (physical) healthcare needs, care givers, and public healthcare professionals with the community of makers and medical herbalists. With the outbreak of COVID-19, the activities of these local hubs, called *Careables Olinda*, completely shifted to producing PPE and other medical supplies in order to fight the pandemic. So far, they have produced around 7,000 face shield units out of which they donated around 5,000 pieces to different initiatives, to hospitals and health authorities in the Recife metropolitan area, cities in the countryside, and indigenous and Afro-Brazilian communities. The remaining products were offered via an e-Shop that was specifically set up for that purpose. The Brazilian fablabs partly relied on shared Open Source designs of face shields and adapted them to the local context. Besides, together with healthcare professionals, they developed an open-source model of an aerosol box integrated into local necessities to use at Intensive Care Units (ICU) and released it online. The aerosol box is used in the process of intubation and extubation of patients, to avoid the contact of aerosol sprays of patients with doctors. The product was validated with doctors from two different hospitals in the Metropolitan Region of Recife, who have experience in orotracheal intubation. In addition, a community including professors, medical students and designers from Casa Criatura was established to develop the product further and is, at the time of writing this manuscript, seeking certification and registration of a free patent, with the final aim of bringing the aerosol box to a global market.

Partnerships and networks, local and global, played a crucial role in this case. Since the beginning of its activities, *Careables Olinda* had a dialogue with the Secretary of Health of Olinda, who realized the importance of the maker community in the local production of PPE to keep up the city’s health system. Later, collaboration with healthcare professionals and hospitals started as ICU professionals approached the makers with a request for a better version of an aerosol box and jointly they defined the specifications. The relationship with the health sector has allowed *Careables Olinda* to review its area of activity and has expanded the scope of its inventiveness while at the same time it increased local awareness and knowledge about open-source, digital manufacturing and healthcare across the involved stakeholders, e.g., physicists, designers, healthcare professionals, makers. The local maker hubs also felt like being part of a bigger initiative, not only for the producers of PPE. Their communication campaign was a small part of a big operation by different sectors in the cities to suppress the virus and the cooperation with the Secretary of Health has already split over to other activities of open innovation, besides COVID-19.

While the activities clearly showed valuable impact in reducing the curve of COVID-19 infections in the regions (contrary to the figures at national level) and in promoting the social value of open innovation, the actors clearly recognize the negative impact of their actions. The enormous amount of plastic being produced, the considerable degree of pollution and carbon emissions related to the makers’ activities, coupled with the environmental problems of the local communities triggered a commitment for more sustainable practices in the future. Some of those have already started to emerge, such as the work with recycled plastic or the creation of a bio-fermentation lab to address the food scarcity in some local communities.

### The Cameroonian Case (Mboalab, Mbankomo)

Mboalab is a community biology lab in the central region Yaounde in Cameroon, comprising molecular biologists, biochemists, public communications specialists, microbiologists, and electro-mechanic technicians, who act as educators in the local community to empower the population with the skills to solve their health and environmental problems. During the COVID-19 pandemic outbreak, the lab tried to attack some of the local bottlenecks related to the sanitary situation. The information department at Mboalab accessed the latest information and recommendations from the World Health Organization (WHO), the Food and Drug Administration (FDA), and the Centers for Disease Control and Prevention (CDC), looking for simple formulae for making hand sanitizers and instructions for face masks designs. With their strategy to target the most vulnerable groups and communities (healthcare workers and the local population) of the suburban community of Mbankomo, where the lab is located, they started their educational work. The lab team was able to prepare alcohol-based hand sanitizers that met the FDA recommendation with locally available components and started demonstrating simple formulae for producing hand sanitizers from cheap and readily available components from the local drug stores.

The lab also produced face masks using appropriate local fabric and prototyped an automatic gel and water dispenser to limit the spread of the virus and encourage frequent washing of hands. Educational aspects were embedded in most activities of the lab. Through the use of the automatic gel dispenser the local population was taught about the importance of handwashing and how an effective hand washing exercise should be carried out. These sensitization sessions were also used to educate the population about the Coronavirus, its modes of transmission, ways of prevention–helping to do away with certain myths about the virus that circulated in the local community.

Again, global networks were important to connect with other populations to share stories, knowledge, and approaches using platforms like Openair and Wikifactory. National and international collaboration was encountered in the search of testing kits, and by participating in the development and testing of simple, easy to replicate methods of fighting COVID-19 in resource constraint settings.

In spite of all these efforts made, some challenges were encountered. Upscaling was an issue, due to a lack of funds. For instance, the automatic gel dispenser that was prototyped was intended to be kept in at least 10 major centers of the community to encourage frequent washing of hands and demonstrate an efficient hand washing practice. The general increase in prices of all essential and non-essential goods made life difficult for the common local population. The widely spread belief that Africans are naturally immune to the virus and that some concoctions can provide them stronger immunity and protect them from being infected was another hurdle. Part of the population also strongly believed and went about saying that the Coronavirus is not real and that it was only a scam or some “thing” created to deceive and control people’s lives.

Given the sensitizations, training, and collaborations achieved during this period of the pandemic, the population, community biologists, and makers stand a chance of independently handling future pandemics or epidemics by confidently producing PPE or other materials that might be required to fight the pandemic. The approach is to educate and equip the population with the skills to be able to handle the crisis without depending on the government, non-profit-organisations, or foreign aiders.

### The Dutch Case (WAAG, Amsterdam)

The maker space at Waag aimed to provide support in maker research, product development and prototyping during the first lockdown phase, teaming up with a nationwide group of Technical Universities, the TechMed Center (University of Twente), the police, the Royal Netherlands Army and national and global maker communities.

In an attempt to better understand the needs within the medical field efforts were mostly dedicated to coordinating and backchanneling within the network. Via online meetings Waag functioned as a catalyst in bringing different maker groups together and has been connecting stakeholders that weren’t in contact or collaboration with each other. It was important to get an overview of products or prototypes that were needed most, but also looking into existing solutions or solutions that were being developed, and how Waag could be involved in this. One of the main concerns and also the main challenge for the Waag team was to ensure the safety of the PPE. Also, getting prototypes tested by certified bodies was difficult. Based on the experiences from other makerspaces in the Netherlands and internationally, door handles and face shields were mostly considered for production.

In collaboration with the police Waag explored the prototyping of door handles, which police officers would use when entering an unknown building. Different production methods were explored to find an alternative for the prototype the police were using, which was 3D printed and consumed quite some time for large scale production. The team at Waag adjusted the model so it could be laser cut, and thus be produced at large scale in a short time. The prototype was tested and functional. However, within the police force few police officers wanted to use a door handle in their daily work. So, the adapted design was in the end not produced. Other products the fablab was experimenting with include a transparent face mask and a DIY respirator.

There was a lot of media attention on the lack of PPE for healthcare professionals in the Netherlands. Waag reached out to the medical institutions within the Metropole region of Amsterdam to hear what their needs and wishes were, but in the end the first contacts ended in no specific request for further research or prototyping. Thus, the fablab at Waag started to produce face shields and opened a webshop to sell them at minimal costs to local healthcare professionals, organizations and people working in contact jobs, such as hairdressers, cleaning services, beauticians, nail salons etc. Local residential care organizations that were directly offered these face shields did not show any interest.

Overall, the impact of Waag’s engagement has been mainly in coordinating and pushing the notion of maker skills towards other domains, rather than organizing and producing PPE. Building on a network of local, national and international organizations, Waag was able to push the added value of maker skills as a driving innovation force. However, when it comes to the production and distribution of large numbers of products Waag was expecting more collaboration with large enterprises, which was not achieved. According to their experience commercial companies are reluctant to take up on the innovative designs and knowledge stemming from the maker communities.

Finally, it is worth mentioning that the discussion within the national coordination group, including the Netherlands Royal Army, also touched upon the notion of distributed manufacturing. The idea of setting up a global network of decentralized production facilities, as an immediate response to a healthcare crisis, gained wider attention and will be further discussed in the future. Currently the network of fablabs mostly shares knowledge, skills and blueprints, but could it also be equipped to coordinate a large-scale production of e.g. PPE in the future?

### The Iraqi Case (Science Camp, Basra)

Science Camp, a maker space based in Basra, south of Iraq, is attached to the global maker movement and used to provide innovative solutions by implementing digital fabrication and DIY concepts, armed with the qualified industrial infrastructure. This space was among the first entities in the country that responded to the COVID-19 crisis with innovative solutions. Approximately 13,000 protective face shields were produced and distributed for free to the frontline medical staff and other main human resources who provide essential services for healthcare, security, delivery services, etc. In collaboration with local industry, local civil society, and academicians, a response infrastructure was set up taking care of e.g. monitoring the needs of PPE, providing raw materials, communicating with healthcare services, PPE production, PPE distribution, online digital statistics monitoring, and research and development. Medical staff highly appreciated the efforts done by the maker community and requested even more face shields and research into other types of PPE.

The design and production process of PPE was adapted to the local context, using locally available raw materials, such as PET plastic sheets used in water packaging factories. Also, the design was adapted to be easy to assemble, with no need for gluing, stapling, or sewing. The digital fabrication techniques applied made the production process use a minimal number of raw materials, and fast, with high quality.

All efforts were covered by voluntary contributions from all partners. The raw materials were a donation from the local water factory. The Iraqi government did, however, not support this type of community response and some international NGOs suggested converting the PPE production process into a business rather than a charity crisis response, which was not realized by the maker space due to ethical reasons.

Apart from the financial challenges, the Iraqi maker space also encountered other difficulties, related to public administration and logistics. Travel permission forms to procure raw materials, machine maintenance, etc. were partly refused during national lockdown and bureaucratic barriers hindered the distribution of PPE via the official channels of the healthcare authorities.

In addition to the high recognition in local and global media, the involved makers got experience in PPE production and legal aspects related to it as well as better insight into the use and availability of raw materials and resources locations. The fast response activities have also shown that the bottom-up social response can work independently from governmental or international aid organizations, avoiding potential conflicts between these organizations.

### The Italian Case (Opendot, Milan)

Italy was Europe’s first and one of the most affected nations being hit by the COVID-19 pandemic. The country’s most efficient health systems in the Northern regions were about to collapse and hospitals were running out of supplies, including PPEs as well as essential parts for ventilators and other respiratory devices. Triggered by the initiative of a maker who provided a hospital with a 3D printer and helped to reproduce missing valves, the value of the maker community for the local health infrastructure became visible, and local supply chains of PPE for healthcare staff and other essential workers started to emerge.

Local coordination groups played an important role in the distributed production and supply chains. These groups were almost all volunteer-based, almost always within existing communities of people or fablab networks who were already used to working together. The first COVID-19 maker networks in Italy were regional, and they succeeded in responding to local needs as they evolved. This teamwork at local and regional levels paved the way for nationwide coordination across Italy. In the period of just a few days, three different initiatives emerged with similar and complementary objectives. One of those initiatives alone, *Make in Italy* ^7^, collected over 500 contacts from makers, small laboratories, startups and fablabs. Their website currently lists over 25,000 items produced and donated. Opendot, a fablab in Milano, *Careables* partner and specialized in working with the healthcare sector, was involved in the national coordination activities from the onset and contributed to overcoming the local medical supply shortage.

Italy’s response to the health crisis wasn’t limited to the grassroots maker movement of hundreds of volunteers and fablabs. Many Italian companies worked closely with active makers, and in some cases, even helped the movement to take off. Also the educational sector was involved as the face shield production model was included in the training of high school students, initiated by the Maker@Scuola project^8^.

The case in Italy has shown so far how new local collaborations for digital social innovations can be established. Because of the emergency, various hospitals contacted specialized studios, fablabs, small businesses and startups in order to develop new solutions together. Doctors have started to become co-designers, innovators and makers. In Italy, we find some isolated examples where these emergency-driven collaborations have turned into established collaborations where hospitals, doctors and therapists recognize the value and potential of digital fabrication tools and distributed local production. However, the organizational and structural details of cooperation between makerspaces and the public healthcare system in a more systematic way are still to be explored.

## Focus Group Results

In the following chapter the results from the focus group discussion with case representatives from Brazil, Cameroon, and Italy are presented.

**FIGURE 4:**
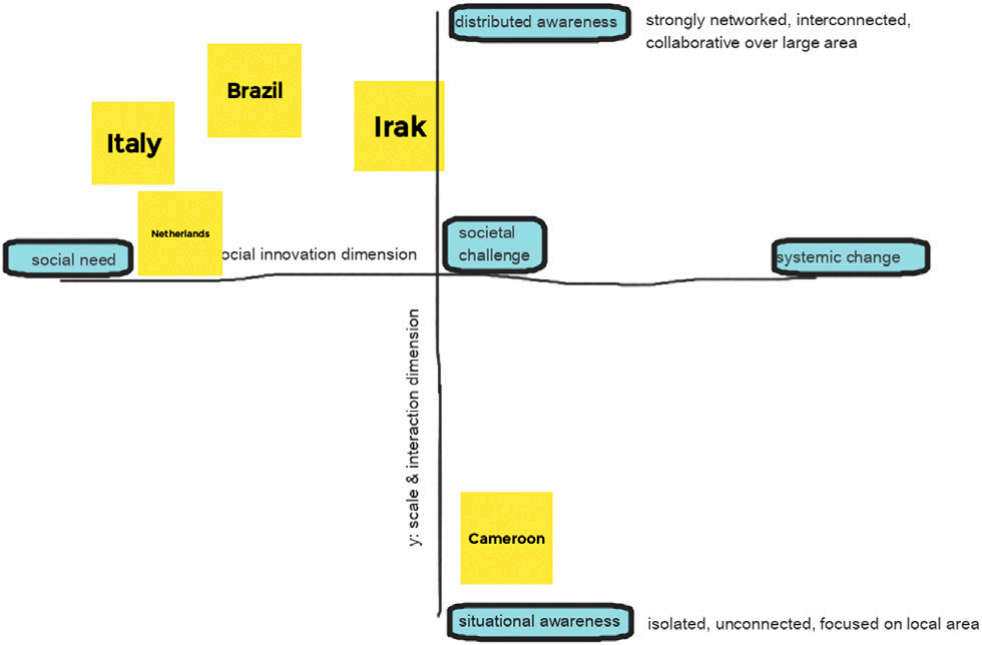
Case positioning on scale and social innovation matrix.

In a first step, focus group participants were invited to relate their Covid-19 activities to one of the levels of social innovation–the micro, meso, or macro level (see Figure 4). The two case representatives not present during the focus group were given an individual explanation of the matrix and were likewise asked for a positioning.

All three case representatives present during the focus group meeting stated that their COVID-19 related activities were in transition from micro to meso level. Thus, cooperation with other organizations became important to meet the social need and not only social but also economic objectives were addressed. The Italian case representative said to have acted on a meso level during the first wave of COVID-19, establishing networks between Italian fablabs and organizations in the need for fabricated health devices; but acted again on a micro level when the emergency situation stopped. Connections with political actors have been established but sustainable links are not in place yet. In the Brazilian case, first sustainable contacts were established with local politicians, who showed interest in the civic engagement taking place in Olinda. Also, the close cooperation with health professionals is still in place, after the first wave. In economic terms, the fablab sells individual face shields, fabricated in their fablab, or whole packages, where e.g., a company is donating 200 face shields. In the Cameroon case the activities were and still are closer to the meso level, as sustainable links to other organizations have been established, mostly related to the educational purpose of the lab.

In a second step, the focus group participants discussed two key questions: 1) If they would wish for their COVID-19 activities to sustainably scale from micro, to meso, to macro level; and 2) if yes, under which conditions.

All three case representatives shared the same opinion–that scaling their activities at least from the micro level to a more stable macro level is wished for, as there are people in the need of help and this need can be met by the production capacities in fablabs. However, scaling up should take place only under certain conditions and building on certain shared principles and values.

Scaling up on the social innovation model should not resemble the scaling up in business terms. It was stated that companies tend to change when they scale, losing contact with local communities, introducing intermediate layers of management, and shifting the focus to financial aspects and maximizing incomes. The risk is that open innovation is not open anymore, but rather owned by a company, thus hindering the innovative groundwork that was originally aimed for by their inventors. So it’s fundamental to change the models of scaling up.

Case representatives wish to scale the approach and spread the specific knowledge on how to co-design and produce (personalized) healthcare devices with digital fabrication tools. So training other fablabs in how to support health and care is key here, but also the training of local people–first regarding COVID-19 and how to best protect themselves, and second how to cooperate with and make use of fablabs to support their health and well-being.

Participants aim for establishing connections and collaboration with other fablabs to scale responses to healthcare professionals and people in need. The Italian experience in this regard shows that creating structured networks of cooperation increases complexity. Fablabs are heterogeneous, have different underlying organizational models and specializations. So not every fablab would be able to produce medical equipment that might work properly in a hospital context. The question is how to deal with this complexity and also raises some doubts that strong networks might result in decreasing visibility and importance of the single node of this network.

Legislation issues have to be addressed carefully if medical equipment is the focus of digital fabrication. When producing healthcare products or medical equipment the main aim is to not produce more harm than good. In Cameroon, only certain institutions are allowed to produce medical equipment, thus Mboalab focused on producing products where no strict legislative measures need to be addressed, e.g., producing and making accessible proper disinfectants and providing the knowledge on how to protect oneself from COVID-19. In Brazil, legislation is more flexible, and the close cooperation with health personnel allowed the fablab in Olinda to successfully develop face shields used in medical organizations as well as an open-source model of an aerosol box. Nevertheless, *Careables Olinda* stresses that working for other groups in the need of health and care, might be a good way to strengthen their approach while avoiding complex certification issues. In the Italian case, the emergency situation of the first COVID-19 wave gave room for certain legislative exceptions. Still, producing for hospitals requires meeting high quality criteria and demands for additional aspects like documentation, etc. In times of crisis, the official processes might be too long and exclusive to flexibly react to emergencies, so there is a call for more agile mechanisms that are established and tested beyond the times of crisis. It is wished for more flexibility to test the collaboration between medical institutions and local manufacturers to co-design and produce medical equipment below certain risks or costs. As mentioned above, working with and for disabled people, and offering COVID-19 support that is not medical equipment (e.g. disinfection, etc.) are some of the suggested activity areas that can strengthen sustainable cooperation between local manufacturers and people and organizations in need of health and care devices. Alternative approaches to supporting health and care are key to spread the approach.

Additionally, strong local nodes are key to spreading the approach. Working in the health and care sector requires at the one hand trust of people and a diverse set of local organizations, on the other hand it aims to successfully empower local people. Thus, establishing a network of local nodes that adapt to local contexts, link to local organizations and the local community of people in need, is the basis of spreading digital fabrication for health and care.

The COVID-19 emergency situation showed how powerful the network of local digital manufacturers can be in flexibly supporting societal needs. Focusing this power of the network to other aspects, like climate change, digital fabrication initiatives can play a key role in successfully supporting social innovation processes. Maker activities can in this regard catalyze the attention to a wider problem, e.g., the climate change and the scaling up should not take place at the cost of the environment. Thus, producing tons of plastics (e.g., in the case of face shields) should be critically reflected and alternative ways of more environmentally friendly production should be sought.

## Summary of Findings

**FIGURE 5:**
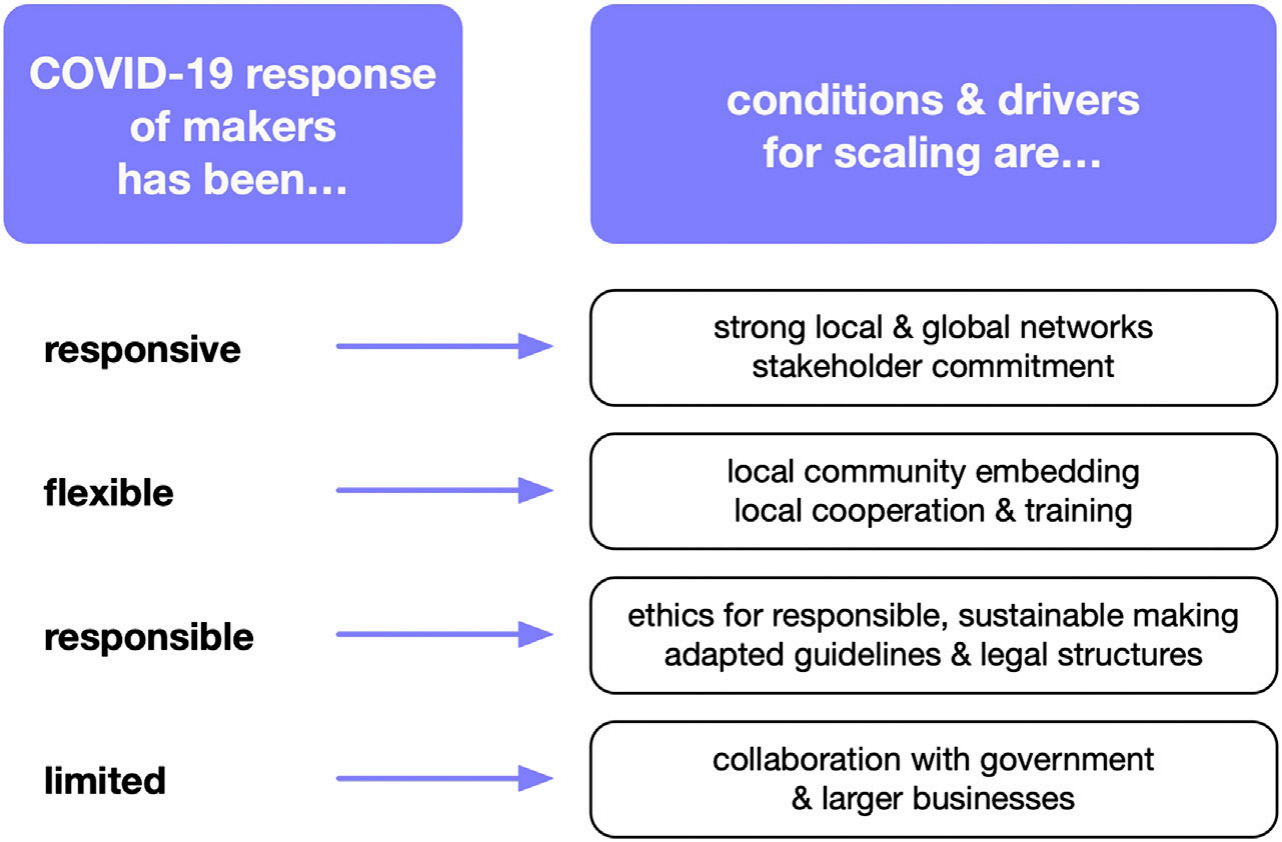
COVID-19 maker response and learnings.

The two main data collection instruments brought forward complementary findings. While the self-reflection reporting focused on describing the past and current experiences made during the COVID-19 response activities by the makers, the focus group discussion built on these experiences and reflected on future implications for the scaling of the makers’ grassroots initiatives. Figure 5 summarizes the experiences made by the cases and the learnings and implications these experiences reveal for a potential scaling in the future.

From the collected experiences we see how fast the maker community reacted during the health emergency by supplying local healthcare providers with urgently needed PPE and other medical devices. Such **responsive** behavior was strongly enabled by the commitment of local stakeholders on the one hand and the global connectedness on the other hand. By drawing from their local, national and global networks the actors in the makerspaces were able to get rapid access to design templates, material resources and distribution channels. Some of the connections with relevant stakeholders, such as medical staff or local politicians, proved to be rather fragile though. For scaling locally embedded maker activities that address local social needs a strengthening of networks, locally and globally, is important according to our cases.

The COVID-19 response of the makers was also **flexible** in adapting to the local contexts. This resulted in localized designs, such as the Brazilian tropical face shield, which was a local adaptation of a German design, or the use of locally available materials, such as the Iraqi PET plastic sheet, which are usually used for water packaging. In the case of the Cameroon maker space the COVID-19 response activities included a strong educational aspect acknowledging the local need for more information about the virus. Next to the importance of education and training on maker skills, the cases also highlight the strong local embedding and connectedness to the local communities as essential for a flexible and fast response to pressing social issues. For a future scaling of maker space activities that respond locally to social needs established cooperation with local stakeholders has been identified as a key element.

Another important aspect addressed in the self-reflection and the focus group discussion has been the awareness for a **responsible** practice from makers. Across the five cases we observe a strong sense of social responsibility. This has been manifested in acknowledgment of quality standards for healthcare products as well as the overall commitment to doing no harm and user safety. Clear guidelines and legal structures that allow responsible making are thus being requested based on the current experiences. When discussing ethical aspects, the case representatives also stress their growing awareness towards the need for ecologically sustainable maker practices.

Finally, the case representatives are aware of the **limitations** of the approach. Their COVID-19 response activities were limited in terms of capacities, resources, and infrastructures. The makers thus recognize the boundaries of what can be achieved within their limited spaces. Collaboration with established businesses and governments should be envisioned for the future. This would allow for an effective response at a larger scale.

## Discussion

The five cases represented in this study are located across three continents and are embedded in very different contexts, economically, politically, and socially. When the COVID-19 pandemic started to spread, these makerspaces took on the challenge of counteracting the PPE and medical device shortages. In their civic reactions to the failures of public healthcare procurement and shortages in the global supply chains, they all faced some similar challenges and new opportunities. In this analysis, we want to concentrate on four main perspectives that evolved during the analysis and turned out to be relevant when discussing the maker COVID-19 response activities in the theoretical frame of the three social innovation levels: social needs (micro level), societal challenges (meso level), and systemic changes (macro level).

1. A Network Perspective

Working in translocal networks, referring to networks at local and global level, has been a critical aspect for the operation on the ground. We saw that reflected in all of the cases, with local networks playing a key role in all phases, from research and design to the production, testing and distribution of the provided COVID-19 response solutions. The established local networks and temporary partnerships include stakeholders from across the quadruple helix, namely academia, government, civil society, and industry. In addition, in most cases, the fast reaction from the makerspaces was only possible due to the global open sharing of PPE designs. Being globally connected offers access to a wide range of resources and is possible only in a culture of openness and sharing, which is propagated also by the Open Design and Open Hardware movements. The benefits of open networked collaborations become visible immediately in times of crisis, such as the COVID-19 pandemic. In social transformative innovation theory translocal networks are an important element contributing to empowerment (Avelino et al., 2019). From the reported experiences in the five cases, we can consider maker communities as local and global networks that exchange resources, experiences, and knowledge at global level, but act at local level to adapt to the specific contexts and react to local needs. This ability and commitment for open global collaboration and mutual learning is described as one of the unique features of the global maker movement (Smith, 2017) and also implies a certain ethical commitment of the contributing makerspaces. The cases contributing to this study confirm their potential for empowerment as suggested by (Avelino et al., 2019) and resilience of the local actors, similar to what has been encountered by Wuyts et al. (2020).

The sustainability of the emergent translocal networks is however very fragile. The Italian case showed that highly efficient and quickly established networks might become loose when the emergency situation is over. For the networks to continue and possibly lead to a transformational change as described by Avelino et al. (2017) new objectives for collaboration that foster a sustainable linkage between network members are needed. For future emergency situations these flexibly emerging translocal networks and partnerships, that have already been installed in previous situations, might help to react even faster.

(2) A Value Perspective

An ethical commitment of the makerspaces becomes noticeable also in other aspects. When discussing ways to scale their practices a need for new types of business models and new value definitions was expressed. Transformative social innovations cannot be achieved by just applying existing innovation models and capabilities to issues of social concern (Smith, 2017). It needs a redefinition of values (Avelino et al., 2017) and a redistribution of innovation capabilities. Globally distributed local manufacturing processes need to be assessed on a different level than large enterprises. Next to the purely economic value, which is still dominating in the entrepreneurial context and also present in many maker space activities, we need to appreciate other values, often social and ecological values, that are associated with local experimentation and small-scale production in makerspaces. The ecological footprint of manufacturing is a concern for many makers as we have seen documented e.g. in the Brazilian case. We can deduct that we find within the activist approaches of makers an environmental conscious reflection and self-critical view on their material productive engagement as confirmed by others (e.g. Smith, 2017; Richterich, 2020). Wuyts et al. (2020) likewise recognize the value of maker activities during the pandemic in moving towards a more circular economy in the healthcare sector.

With the presented experiences we argue that environmentally and socially responsible making should be assigned additional value, next to the cost-benefit calculations dominating today’s business models and move to added value-oriented models. The case representatives in this study follow a community-driven approach, not a business-driven approach, which needs higher societal recognition. There are attempts to raise broader attention for the values of transformative social innovations in measurable terms, such as the Social Return on Investment (SROI), which is a performance measurement tool, demonstrating the social value enterprises generate. SROI is however an underused and undervalued practice despite being accepted as an internationally recognized measurement tool for social enterprise (Millar and Hall, 2012). For community-driven approaches in makerspaces new value models for scaling are needed as the makerspaces of this study clearly do not want to follow the prevailing economic model of scale dominated by monetary value.

(3) An Educational Perspective

Makerspaces are often characterized as spaces for collaboration, information sharing, reflection and learning (Sheridan et al., 2014). Incidental as well as intentional learning takes place in these settings as they are often linked with creativity, collaborative problem solving, digital competence, and entrepreneurship (Vuorikari et al., 2019). An educational agenda was stressed in the Cameroon case, where an important objective of the lab’s COVID-19 response activities was to educate the local population in terms of hygiene measures. The other case representatives emphasized the importance of education in their activities generally, and specifically in terms of scaling social innovations.

Also, learning between makerspaces and with and from other network partners, like health professionals, is key. We see knowledge exchange on a global scale that addresses overarching topics, like the exchange of certified, proven PPE instruction guides, guidelines on the design of co-creation processes, and the efficient use of digital fabrication tools. And we can identify contextualized knowledge that emerges in the diverse settings, like how to react to the local availability of material, how to adapt production processes to local contexts, how to address very specific local needs. Undoubtedly, the learning taking place in makerspaces leads to empowerment and resilience (Criado et al., 2016; Unterfrauner et al., 2020). As Ratto (2011) identified learning as core in his critical making theory, where the process of making is as important as the results, we also suggest that more societal recognition could be added to the educational value created in makerspaces. Critical skills acquired during the material exploration contribute to the empowerment of the individuals as well as the community. Again, we see similarities here to the cases of empowerment analyzed in detail by Avelino et al. (2017). Learning and practicing new skills in social spaces are key elements for empowerment and contribute to the transformative potential of social innovations.

(4) A Legal Perspective

In order for local manufacturing to become relevant at a systemic level, fundamental transformations of the underlying structures need to take place. In the context of open healthcare, current legal frameworks are one of the key structures that would require adaptation. As system changes are typically slow and require long-term thinking, makers are exploring the current boundaries in their support of the healthcare sector. Part of the current boundaries being explored by makers relate to the nature of the solutions they produce. In some states (e.g. in the European Union) the production of specific solutions–such as respiratory valves or breathing masks–requires complex processes and compliance documentation. These are necessary as the solutions qualify as medical devices and imply the respect of the relevant laws in the matter (Medical Devices Directive, 1993, in the European territory). While the role of these regulations is to ensure a high level of patients’ safety and protection, they set approval mechanisms and controls that are not always compatible with emergency situations. In some countries, competent authorities allowed for emergency use authorization for certain technologies (Food and Drug Administration, 2020). As Pearce (2020, p. 12) noted, many regulatory roadblocks remain across several countries, which may need to be improved to allow rapid response and provision of medical supplies in healthcare emergencies.

A second kind of boundary relies on liability mechanisms for makers in the context of emergency situations. As illustrated in the introduction, in a known case some makers reverse-engineered the design of a respiratory valve to face a product shortage in an Italian hospital, which led the original manufacturer to threaten bringing legal action against them for intellectual property infringement. This example explicates the difficult value balance between the perceived need to act (even “ethically”) by makers vis-a-vis the possible unintended negative consequences of such ethical acting. As a way forward, so-called *Good Samaritan Laws*–which offer protection from liability for those whom they believe to be in peril, ill, or otherwise incapacitated - could set useful measures to counterbalance this dichotomy. This legal perspective could help reduce the barriers for companies and makers hindering the release of healthcare projects’ designs and their replication. The case of COVID19 opened new scenarios for the application of these laws. We are aware of the complexity of system innovation as they are “profound transformations in social systems” (Grin et al., 2010) and we believe that we are still far from seeing innovations being fully implemented in our current legal systems, but the recent experiences during COVID-19 have started to challenge the current boundaries. Thus, future exploration, both in research and by policymakers is needed (Pearce, 2020) and it would be capable of opening new perspectives for the makerspaces and the role of makers.

## Conclusion

The experience brought forward in our five contextually very different cases has shown how local production networks can function in times of emergency. Their local design, production, and distribution of PPE and other healthcare related products towards health professionals and the general population has proven to flexibly cover emerging needs and stand in for global manufacturers. Looking at the makers’ initiatives during the COVID-19 crisis from a social and transformative innovation perspective, we encounter a wish to scale from working on the social needs level to addressing wider societal demands and, in the future, even triggering systemic change. Networking and sharing knowledge and experiences across multiple actors are key with this aim. Representatives from the five cases stress the importance of emergent translocal networks for their COVID-19 response to happen, which include actors of the quadruple helix on local scale while exchanging and learning from each other globally.

Scaling transformative practices of makerspaces is however envisioned only under certain circumstances and following a set of principles and values. A commitment towards openness and sharing, such as it is propagated by the open hardware movement, requires new forms of business and value models. Prototyping in the healthcare domain, with and for patients, people with disabilities, and other often vulnerable groups, requires an ethical commitment and legal backing in order not to produce more harm than good. Educational and environmental considerations likewise come into play. Empowerment through teaching and creating only solutions that address real personal problems or needs are core principles of responsible making. In the makers’ future endeavors towards co-designing and making open, personalized healthcare and establishing these processes as social innovations more social value propositions may be encountered, with implications for individuals, communities and society at large. While we notice signs of empowerment at individual and community level, we envision a strengthening of democratic processes at society level. Other scholars likewise speak about the democratic value of makerspaces, which they find in certain grassroots activities that address social issues (e.g. Taylor et al., 2016; Willingham, 2017; Sipos et al., 2019). At the same time, we are aware of the critical views some scholars express towards makerspaces. Lindtner et al. (2016) challenge the democratization potential of the maker movement and suggest a more self-critical and reflexive approach for the whole community of makers. We hope this study can contribute to the discussion.

We have considered implications that go beyond the makers’ response to fighting COVID-19 from the experiences made in five contextually diverse settings. We are aware of the limitations of our study, but see reasonable generalization justified by the contextual heterogeneity of the cases covered, the strong embeddedness and connectedness of the case representatives (and co-authors) with the global maker community, and the similarities we have found on other documented cases, such as those documented by others. Our case-based snapshots resonate well with other documented experiences (e.g. Diez and Baeck, 2020; Richterich, 2020). Next to this qualitative approach a more systematic and quantitative assessment of the impact that the maker communities worldwide had on fighting the COVID-19 pandemic is needed. In how far has the global maker response during the COVID-19 emergency situation created sustainable impact and have longer-term linkages between local manufacturers and health care services been created? Also, we would love to see more explorations of how and under which conditions makerspaces contribute to addressing societal challenges and how these may trigger systemic change in the future.

## Data Availability

The raw data supporting the conclusions of this article will be made available by the authors, without undue reservation.

## Footnotes

1
https://www.careables.org/discover-careables/

2
https://www.welder.app/careables

3
https://opensourcemedicalsupplies.org/

4
https://www.globalinnovationgathering.org

5
http://www.opendotlab.it/about/

6
https://waag.org

7
http://www.makeinitaly.org/

8
http://www.indire.it/en/progetto/maker-at-school/
